# Dual-Multivalent Aptamer-Based Drug Delivery Platform for Targeted SRC Silencing to Enhance Doxorubicin Sensitivity in Endometrial Cancer

**DOI:** 10.7150/ijbs.101059

**Published:** 2024-10-28

**Authors:** Haojia Li, Shuangshuang Cheng, Qi Zhang, Ting Zhou, Tangansu Zhang, Shuangge Liu, Yingying Peng, Jia Yu, Jingwen Xu, Qi Wang, Jun Zhang, Yuwei Yao, Hongbo Wang

**Affiliations:** 1 Department of Obstetrics and Gynecology, Union Hospital, Tongji Medical College, Huazhong University of Science and Technology, Wuhan 430023, China.; 2 Clinical Research Center of Cancer Immunotherapy, Wuhan 430023, China.; 3 School of Public Health, Tongji Medical College, Huazhong University of Science and Technology, Wuhan 430030, China.; 4 Department of Dermatology, Union Hospital, Tongji Medical College, Huazhong University of Science and Technology, Wuhan 430023, China.

**Keywords:** endometrial cancer, targeted drug delivery, doxorubicin, rolling circle amplification, ferroptosis

## Abstract

Endometrial cancer poses a significant threat to women's health. Doxorubicin is commonly used in chemotherapy for advanced and recurrent cases; however, low sensitivity frequently limits its effectiveness. In this study, we verified that SRC modulates the sensitivity of endometrial cancer to chemotherapy of doxorubicin and developed a targeted silencing drug delivery platform that employs rolling circle amplification and dual-multivalent aptamers to precisely deliver therapeutics directly to tumor cells. This platform enhanced endometrial cancer cell sensitivity to doxorubicin by modulating drug responsiveness at the genetic level. Our results suggest that this approach may improve cancer cell susceptibility to ferroptosis. The efficacy and safety of this platform were validated in both cellular and animal models. This study provides a new solution for realizing the precision treatment of endometrial cancer and lays a theoretical foundation for exploring the mechanism of endometrial cancer.

## Introduction

Endometrial cancer (EC) has the highest global incidence among the three gynecological malignancies^1^. Its prevalence and mortality are persistently increasing^2^. In 2023, the International Federation of Gynecology and Obstetrics defined four molecular subtypes of EC based on The Cancer Genome Atlas (TCGA): DNA polymerase epsilon (POLE) ultramutated, microsatellite instability/high mismatch repair deficiency (MSI-H/dMMR), copy-number low (CNL), and copy-number high (CNH)^3^. These classifications mitigate the constraints of the previous Bokhman classification system. Although patients with early-stage EC have a favorable 5-year survival rate exceeding 80%, those with advanced EC face shortened survival times and a lack of therapeutic options^4^. Standard first-line treatments for advanced and recurrent EC include combination chemotherapy, particularly carboplatin/paclitaxel regimens^5^. Furthermore, immune checkpoint inhibitors (such as PD-1/PD-L1 inhibitors) have improved efficacy, particularly in MSI-H/dMMR EC^6^. Despite the continuous emergence of novel therapies in recent years, the prognosis of patients with advanced or recurrent EC remains poor.

Doxorubicin (DOX), a member of the anthracycline class of cytotoxic agents, effectively halts the progression of various malignancies, including leukemia, breast cancer, and ovarian cancer^7^,^8^. The anti-neoplastic mechanisms of DOX are multifaceted. Within the cytoplasm, the quinone moiety of DOX acts as an electron acceptor, engaging in redox reactions that generate reactive oxygen species (ROS) through intracellular metabolism. The induction of ROS results in oxidative stress, lipid peroxidation, and membrane and DNA damage, causing multiple forms of cellular damage^9^-^11^. Within the nucleus, DOX disrupts topoisomerase activity (specifically topoisomerases I and II) by intercalating into the double-stranded DNA helices. This interference hampers replication and transcription, thereby curbing DNA and RNA synthesis in rapidly proliferating cells^12^,^13^.

A systemic chemotherapy regimen combining DOX and platinum-based agents is recommended for advanced and recurrent EC. Single-agent chemotherapy may be a suitable alternative for patients who are intolerant to combination chemotherapy^14^,^15^. However, DOX use in cancer therapy has encountered significant obstacles. Drug efflux mechanisms (such as ATP-binding cassette transporter family member overexpression in cancer cells) contribute to resistance or reduced sensitivity to various chemotherapeutic agents, including DOX^16^,^17^. In contrast, the suppression of cell death pathways (including autophagy and apoptosis) within tumor cells can also reduce the therapeutic efficacy of DOX^18^,^19^. Therefore, investigating the mechanisms underlying the reduced sensitivity of tumors to DOX during cancer treatment is important for enhancing chemotherapy efficiency and efficacy. Additionally, DOX induces apoptosis and necrosis in normal tissues, resulting in toxicity to organs such as the brain, liver, and kidneys, with life-threatening cardiotoxicity being particularly significant. These adverse reactions majorly limit DOX use in chemotherapy^20^,^21^. Hence, enhancing DOX targeting is a key strategy to address its severe adverse effects.

DNA nanotechnology is an emerging field that harnesses biomolecule self-assembly properties. It has garnered attention because of the predictable secondary structure, small size, biocompatibility, and programmability of DNA, offering wide-ranging applications in nanomaterial construction, nanorobotics, drug delivery, molecular sensing, and nanoelectronics^22^,^23^. Within the Watson-Crick base-pairing programming environment, hybrid DNA strands can be designed to form highly spatially-programmable functional nanostructures and provide chemical sites for biomolecule functionalization, thereby serving as carriers for targeted applications. Recently, DNA nanomaterials have been extensively studied. DNA origami enables the folding of long single-stranded DNA (ssDNA) molecules by synthesizing short complementary DNA segments, providing a simple yet powerful tool for the precise control of structures at the nanoscale^24^,^25^. However, DNA origami also faces limitations, such as constrained conformational changes and reliance on strand displacement reactions^26^. DNA hydrogels consist of hydrophilic polymer networks cross-linked with DNA strands. Functionalized hydrogels prepared with aptamer modules have improved sequence programmability and molecular recognition capabilities, enabling efficient anticancer drug loading and integration of cancer treatment effects. This facilitates targeted drug delivery and controlled drug release, which are advantageous for cancer therapy^27^,^28^. Nucleic acid aptamers form unique three-dimensional folds through intramolecular interactions, generating ssDNA or RNA molecular structures that can bind to substances, such as proteins, peptides, and metal ions^29^. Advantages include binding with high selectivity and affinity, ease of chemical modification, rapid tissue penetration, low immunogenicity, low toxicity, and small size^30^. Therefore, aptamers are widely studied and applied in cancer diagnosis and treatment. Additionally, aptamers exhibit thermal stability and self-recovery, denaturing at 95°C, but refolding into the correct three-dimensional conformation at room temperature, making them easy to assemble with other materials^31^. Thus, the organic integration of aptamers with DNA nanomaterials offers new avenues for targeted anti-tumor therapeutic strategies. However, relevant studies using DNA nanotechnology to target endometrial cancer are still few.

SRC is a non-receptor tyrosine kinase that is activated by multiple signaling pathways. Once activated, SRC phosphorylates the tyrosine residues of target proteins, thus activating the corresponding signaling pathways, including the MAPK, STAT, PI3K/AKT, and EGFR pathways. Aberrant SRC protein activation is associated with various tumors, and SRC activity is closely correlated with tumor progression. Notably, the SRC signaling pathway plays a pivotal role in chemotherapy resistance^32^. In osteosarcoma and colon cancer, a combination of SRC inhibitors enhances tumor sensitivity to DOX therapy^33^,^34^. In EC, SRC is hyperactivated across multiple biological pathways, affecting EC cell sensitivity to DOX^35^.

In this study, we aimed to develop a targeted drug delivery platform utilizing dual-targeting and multivalent aptamers to modulate the sensitivity of EC cells to DOX. Our experimental results confirm that SRC expression is a critical pathway affecting the responsiveness of EC cells to DOX treatment. This pathway may operate via ferroptosis regulation in EC.

## Materials and Methods

**Materials.** All DNA and RNA used in this experiment were synthesized by Sangon Biotech (Shanghai, China), and the sequences are listed in Table S1. T4 DNA ligase and dNTP Mixture were purchased from Sangon Biotech (Shanghai, China). Phi29 DNA Polymerase was procured from Beyotime Biotechnology (Shanghai, China). Doxorubicin was obtained from MedChemExpress (MCE).

**Cell Culture.** Human endometrial cancer cell lines AN3CA, KLE, Ishikawa, HEC-1A, HEC-1B, and RL95-2 were purchased from Wuhan Pricella Biotechnology Co., Ltd. KLE, HEC-1B, and Ishikawa cells were cultured in DMEM/F12 medium (Gibco) containing 10% fetal bovine serum (FBS). RL95-2 cells required an additional 5 μg/ml insulin, while HEC-1A cells were cultured in McCoy's 5A medium containing 10% FBS. AN3CA cells were cultured in MEM with 10% FBS. All media were supplemented with 1× penicillin-streptomycin solution (100U/ml penicillin, 0.1mg/ml streptomycin, 1% P/S, Procell). Cells were incubated at 37°C in a 5% CO_2_ atmosphere.

**RNA and Protein Extraction.** According to the manufacturer's instructions, total RNA was isolated from cells or tissues using the TRIzol™ Plus RNA Purification Kit (Thermo Fisher Scientific). Membrane proteins were extracted using the Mem-PER™ Plus Membrane Protein Extraction Kit (Thermo Fisher Scientific). For total protein extraction, cells were lysed with RIPA lysis buffer (G2002, Servicebio), and the extracted total protein was quantified using a BCA assay kit (Biosharp). Proteins were denatured by adding 5× loading buffer (AR1112, BOSTER) and boiling for 8 minutes.

**Isolation and Culture of Primary Cells.** The isolation of normal endometrial glandular epithelial cells was primarily based on the method by Zhang^36^, with some modifications. Ethical approval number: [2021] S046. First, under sterile conditions, 3-5 g of endometrial tissue was scraped and placed in DMEM/F12 medium containing 1% P/S and metronidazole. In a biosafety cabinet, the tissue was washed three times with D-Hank's balanced salt solution containing 1% P/S and metronidazole, followed by three washes with cell culture medium. The endometrial tissue was minced and digested with Type II collagenase at 37°C for 8-10 minutes. Digestion was stopped with twice the volume of DMEM/F12 medium, and a cell suspension was prepared. The cell suspension was filtered through a 100-mesh cell strainer to remove mucus and undigested tissue. The filtrate was then filtered through a 400-mesh cell strainer, and the strainer was washed with culture medium to collect cell clumps on the strainer surface. The cells were centrifuged at 1000 rpm for 10 minutes, and the cell pellet was seeded into culture flasks and incubated at 37°C in a 5% CO_2_ incubator. The primary cell extraction from endometrial adenocarcinoma was similar to the above method. After obtaining the digested cell suspension, it was filtered through a 100-mesh cell strainer, centrifuged at 1000 rpm for 5 minutes, the supernatant was discarded, and the cell pellet was seeded into culture flasks under the same conditions as the endometrial glandular epithelial cells.

**qRT-PCR.** According to the manufacturer's instructions, reverse transcription was performed using ABScript III RT Master Mix (RK20428, ABclonal) with the following program: 55°C for 15 minutes, 85°C for 5 minutes, and then hold at 4°C. The resulting cDNA was amplified using Universal SYBR Green Fast qPCR Mix (RK21203, ABclonal), and the fluorescence signals were monitored in real-time using a CFX Connect™ Real-Time PCR Detection System (Bio-Rad).

**Western Blotting.** Proteins of equal quality were separated by SDS-PAGE at 120V for 90 minutes and transferred to a PVDF membrane (ISEQ00010, Merck Millipore) at 280mA for 90 minutes. The membrane was then blocked in 5% non-fat milk (GC310001, Servicebio) for 1.5 hours. Primary antibodies were incubated overnight at 4°C, followed by incubation with the corresponding secondary antibodies at room temperature for 1 hour. Protein bands were detected using ECL reagent (BL520A, Biosharp) and the BG-gdsAUTO 710 MINI chemiluminescence imaging system (Baygenebiotech). The primary antibodies used in this study were SRC (11097-1-AP, Proteintech), KI67 (27309-1-AP, Proteintech), NCL (PAB34311, Bio-swamp), TFRC (PB9233, BOSTER), ACSL4 (PAB34846, Bio-swamp), STAT3 (10253-2-AP, Proteintech), and p-STAT3 (BM4835, BOSTER). The secondary antibody was HRP-conjugated Goat anti-Rabbit IgG (H+L) (AS014, Abclonal).

**IC50 Detection Experiment.** Cells were seeded at 100 μL per well in a 96-well plate and incubated in a cell incubator (37°C, 5% CO_2_). For the cells to be tested, 1/10 volume of Cell Counting Kit-8 (C0005, TargetMol) was added directly to the culture medium, mixed thoroughly to ensure uniform color without bubbles. For each 100 μl of culture medium in the 96-well plate, 10 μl of detection reagent was added. The cells were further incubated in the incubator for 1-4 hours. Before reading with a microplate reader, the plate was shaken on a shaker for about 1 minute to ensure uniform color. The absorbance was read at 450 nm to calculate cell viability. IC50 values were calculated using the Nonlinear Regression-Dose-Response-Inhibition of GraphPad Prism 9.4.0.

Cell viability = [(Experimental group - Blank group) / (Control group - Blank group)] × 100%

Cell inhibition rate = [(Control group - Experimental group) / (Control group - Blank group)] × 100%

**Cell Proliferation Experiment.** Cells were incubated with 1× EdU for 2 hours according to the instructions provided with the BeyoClick™ EdU-488 (C0071S, Beyotime). Cells were fixed with 4% paraformaldehyde for 15 minutes, the fixative was removed, and cells were washed three times with PBS for 3 minutes each. After removing the washing solution, PBS containing 0.3% Triton X-100 was added for cell permeabilization and incubated at room temperature for 15 minutes. The permeabilization solution was removed, and cells were washed twice with PBS for 3 minutes each. The Click reaction solution was prepared according to the instructions and added to the wells for 30 minutes. Hoechst 33342 was diluted 1:1000 with PBS and incubated at room temperature in the dark for 10 minutes, followed by three washes. The cells were then observed under a fluorescence microscope. For cells with >90% adhesion rate (in logarithmic growth phase), cells were digested, centrifuged, resuspended, and counted. For a six-well plate, 1000 cells were added to each well and incubated at 37°C in a 5% CO_2_ incubator. When colonies became visible to the naked eye, the six-well plate was removed, the medium was discarded, and the cells were washed with PBS. Each well was fixed with 1 ml of 4% paraformaldehyde for 30 minutes, washed with PBS, and stained with 1 ml of 0.1% crystal violet for 10 minutes. The wells were then washed with PBS, and colonies were photographed and counted using a digital camera.

**Wound Healing Experiment.** The day before the experiment, cells were seeded in a six-well plate at a density of approximately 5×10^5 cells per well to reach 100% confluence the next day. A scratch was made using a 200 μl pipette tip, the medium was discarded, and the wells were washed before adding drug-containing medium or serum-free medium. Pictures were taken at 0 hours as a control. The cells were then incubated at 37°C in a 5% CO_2_ incubator. At different time points, the cells were observed under a microscope for changes in scratch width and photographed.

**Transwell Invasion Assay.** Matrigel was thawed at 4°C overnight. On ice, Matrigel was diluted to 1 mg/mL with serum-free medium and mixed to a uniform state using pre-cooled pipette tips. 60 µL of this mixture was carefully added to the bottom of an 8 µm Transwell chamber (3422, Corning) to form a uniform layer, avoiding bubble formation. The chamber was then incubated at 37°C for 1-3 hours. 100 µL of serum-free medium was added to hydrate the chamber, and the plate was incubated at 37°C for 30 minutes. After removing the medium, it was checked that no liquid had passed through to the lower chamber, indicating the setup was ready for cell seeding. 500 µL of complete medium containing 10% FBS was added to the lower chamber of a 24-well plate. Using tweezers, the Transwell chamber was placed into the well. Cells treated with the drug were trypsinized, resuspended in serum-free medium, and added to the chamber. The plate was incubated at 37°C with 5% CO_2_ for 24 hours. The Transwell chamber was removed, and the medium was discarded. The chamber was gently wiped with a PBS-moistened cotton swab to remove Matrigel and cells from the inner surface. 300 µL of 4% paraformaldehyde was added to a clean well of the 24-well plate, and the chamber was fixed for 15 minutes. After discarding the fixative, the chamber was washed once inside and outside with PBS. 300 µL of crystal violet staining solution was added to a clean well, and the chamber was stained for 15 minutes. The chamber was washed three times inside and outside with PBS. After air-drying, the cells were observed under a microscope, and 3-5 fields were photographed for quantitative analysis using ImageJ.

**Immunohistochemical Staining.** Tissues were fixed in 4% paraformaldehyde, embedded in paraffin, and sectioned. Paraffin blocks were softened in an ice-water mixture. Sections were baked at 60°C for over 4 hours and then deparaffinized by immersing in xylene and different concentrations of ethanol: 70%, 80%, 90%, 95% for 5 minutes each, and 100% for 10 minutes. Endogenous peroxidase was blocked for 20 minutes. Antigen retrieval was performed by boiling the sections in citrate buffer for 10 minutes. The sections were incubated overnight at 4°C with primary antibodies, followed by incubation with a reaction enhancer at room temperature for 20 minutes, washed with PBS, and incubated with secondary antibodies. Color was developed using DAB substrate. Hematoxylin was used for counterstaining, and sections were blued with an alkaline solution. Dehydration was carried out by immersing the sections in a gradient of ethanol: 70% for 1 minute, 80% for 1 minute, 90% for 1 minute, 95% for 1 minute, 95% for 5 minutes, and 100% for 5 minutes. Sections were cleared with xylene and mounted with neutral resin, then allowed to air dry naturally.

**Preparation of DSNClew.** To prepare the circular template for RCA, padlock (1 μM), primer (1 μM), T4 DNA ligase buffer (1x), and water were mixed. The mixture was heated to 90°C and then annealed at 55°C. T4 DNA ligase (50 U/ml) was added and incubated at 22°C for 3 hours, followed by inactivation at 65°C for 10 minutes. Phi29 DNA polymerase was diluted to 10 U/ml, and 1 mM dNTP and the circular template were added. The mixture was amplified at 30°C for an appropriate time, then inactivated at 65°C for 10 minutes to complete the preparation of DNAclew. DNAclew was mixed with different concentrations of AptL-AS1411 and AptL-tJBA8.1, incubated at 95°C for 5 minutes, then cooled down to 4°C at a rate of 1.5°C/min, and maintained at 4°C for over 3 hours. Subsequently, it was incubated with various concentrations of doxorubicin at 25°C for 2 hours. Finally, OH-siSRC was added and incubated at 37°C for 6 hours to obtain DSNClew.

**Agarose Gel Electrophoresis.** Agarose (A620014, Sangon) was mixed with 1x TAE buffer (B548101, Sangon) and boiled until completely dissolved. After cooling to 60°C, 1x Gelred (A616697, Sangon) was added. The solution was shaken, swirled, or inverted to ensure thorough mixing of the stain. The gel was poured, samples were loaded, and the gel was run at 110V for 40 minutes using the DYCP-32C horizontal electrophoresis apparatus (122-3230, Liuyi Biotechnology). The results were observed and photographed using a GenoSens S2 gel imaging system (Clinx).

**Scanning Electron Microscopy (SEM) and Transmission Electron Microscopy (TEM).** DSNClew was quick-frozen in liquid nitrogen, then dried thoroughly overnight in a vacuum freeze dryer. The dried samples were coated with gold for 45 seconds, and the morphology was captured using a ZEISS GeminiSEM 300 scanning electron microscope with an accelerating voltage of 3kV. The samples were dehydrated with different concentrations of acetone, embedded with embedding medium at 37°C overnight, and sectioned. A copper grid was used to adsorb the sections, stained with uranyl acetate and lead citrate, and dried. The samples were tested using a Hitachi H-7650 biological transmission electron microscope (TEM, Japan).

**Hemolysis Assay.** Mice were anesthetized with 1% pentobarbital sodium, and 500 μL of blood was collected from the eye. 2 mL of PBS was added, centrifuged at 1000 rpm for 5 minutes, and the supernatant was discarded. The process was repeated until the supernatant was clear. The red blood cells were resuspended in 500 μL PBS. For the positive control, 50 μL of red blood cell suspension was mixed with 80 μL H2O. For the negative control, 50 μL of red blood cell suspension was mixed with 80 μL PBS. For the experimental group, 50 μL of red blood cell suspension was mixed with 80 μL of different concentrations of DSNClew. The mixtures were incubated at 37°C for 0.5 hours, then centrifuged at 1000 rpm for 5 minutes.

**Subcutaneous Tumor Xenograft Experiment.** Female BALB/c-nu nude mice (1 month old) from Beijing Vital River Laboratory Animal Technology Co., Ltd. were maintained in a standard SPF environment. HEC-1A cells were suspended in PBS and subcutaneously injected into the left scapula of the mice. Tumor size was measured every 7 days. After 4 weeks, mice were euthanized by cervical dislocation, and the subcutaneous tumors were excised. Tumor weight, length, and width were measured. Tumors were paraffin-embedded for subsequent histopathological analysis. The animal experiment design of this study was reviewed and approved by the Animal Ethics Committee of Tongji Medical College, Huazhong University of Science and Technology ([2022] IACUC Number: 3356).

**Malondialdehyde (MDA) Assay.** Using a lipid peroxidation assay kit (S0131S, Beyotime), the relative concentration of MDA in cell lysates was measured according to the manufacturer's instructions. Endometrial cancer cells (5 × 10^6) were seeded in 10 cm culture dishes and treated with corresponding drugs for 12 hours. The medium was discarded, and cells were washed twice with PBS. Cells were lysed using cell lysis buffer (P0013, Beyotime). After centrifugation at 12000 g for 10 minutes, 100 μL of the supernatant was mixed with 200 μL of MDA working solution and incubated at 100°C for 15 minutes. After cooling to room temperature, the mixture was centrifuged at 1000 g for 10 minutes. 200 μL of the supernatant was taken, and absorbance was measured at 532 nm.

**Reactive Oxygen Species (ROS) Assay.** Using a ROS detection kit (S0033S, Beyotime) according to the manufacturer's instructions, DCFH-DA was diluted to a final concentration of 10 μmol/L with serum-free medium (1:1000 dilution). The cell culture medium was removed, and DCFH-DA working solution was added to cover the cells. Incubate at 37°C for 20 minutes, and then wash the cells three times with serum-free medium. Observe the cells loaded with the probe using a fluorescence microscope.

**Cellular Ferrous Ion Level Detection Assay.** Using an Iron Assay Kit (ab83366, Abcam) according to the manufacturer's instructions, ferrous ion levels in cells were measured. After 12 hours of drug treatment, cells (1 × 10^7) were collected and lysed with buffer (P0013, Beyotime). The cell extracts were centrifuged, and the supernatant was collected. An iron reducer was added to each sample, mixed, and incubated at room temperature for 30 minutes. Then, 100 μL of iron probe was added to each sample, mixed, and incubated in the dark at room temperature for 1 hour. The absorbance of the mixture was measured at 593 nm.

**GSH and GSSG Detection Assay.** Using the GSH and GSSG assay kit (S0053, Beyotime) according to the manufacturer's instructions, samples were prepared for measuring total glutathione content. The cells were washed once with PBS, centrifuged to collect the cells, and the supernatant was removed. Protein removal reagent M solution in an amount three times the cell pellet volume was added and vortexed thoroughly. Two rapid freeze-thaw cycles were performed using liquid nitrogen and a 37°C water bath. The samples were placed at 4°C for 5 minutes, then centrifuged at 10000 g for 10 minutes at 4°C. The supernatant was collected for total glutathione measurement. To prepare samples for measuring GSSG content, a portion of the total glutathione sample was taken and diluted GSH scavenging auxiliary solution was added at a ratio of 20 μL per 100 μL sample. The mixture was vortexed immediately. Then, GSH scavenging working solution was added at a ratio of 4 μL per 100 μL sample and vortexed immediately. The mixture was allowed to react at 25°C for 60 minutes. The absorbance was measured at 412 nm, and the GSH content was calculated using the formula: GSH=Total Glutathione-GSSG×2.

**Mitochondrial Membrane Potential Assay.** Using the mitochondrial membrane potential assay kit (JC-1) (C2006, Beyotime) according to the manufacturer's instructions, the JC-1 working solution was first prepared. The cell culture medium was removed, the cells were washed once with PBS, and an appropriate amount of cell culture medium was added. 1 mL of JC-1 staining working solution was added and mixed thoroughly. The mixture was incubated at 37°C for 20 minutes in a cell culture incubator. During the incubation, an appropriate amount of JC-1 staining buffer (1X) was prepared and placed on ice. After incubation, the supernatant was removed and the cells were washed twice with JC-1 staining buffer (1X). 2 mL of cell culture medium was added and the cells were observed under a fluorescence microscope.

**Data Analysis.** The statistical analysis for comparisons between groups was performed using Student's t-test and ANOVA. Data analysis and presentation were conducted using Prism 9.4.0 (GraphPad Software Inc.).

## Results

### SRC Regulates the Principal Pathways Involved in Sensitizing EC Cells to DOX Therapy

**Figure 1:**
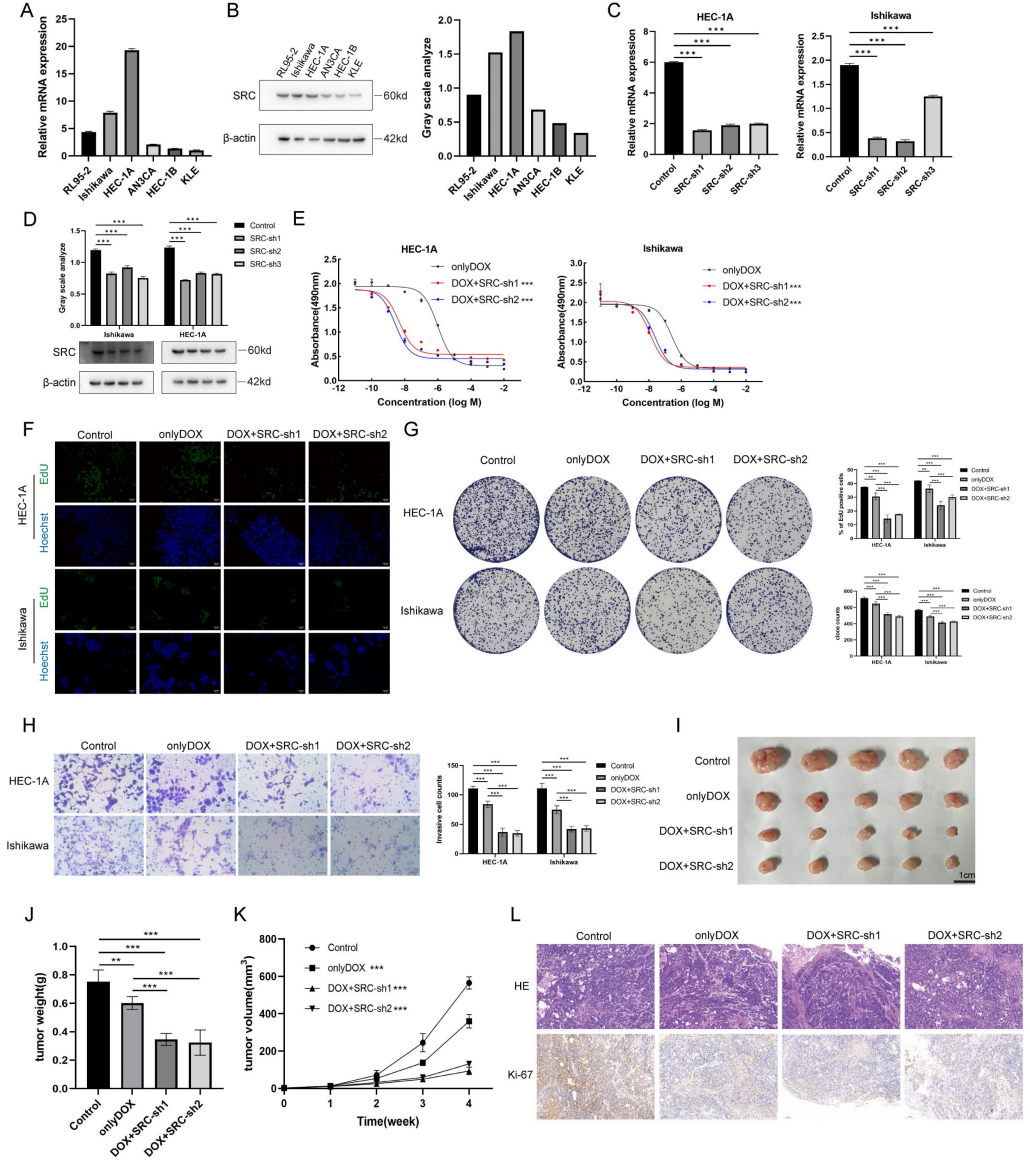
SRC Regulates Sensitivity of Endometrial Cancer to Doxorubicin. (A) Relative mRNA expression levels of SRC in six endometrial cancer cell lines. (B) Immunoblot analysis and grayscale values of SRC protein expression levels in six endometrial cancer cell lines. (C) Relative mRNA levels of SRC in EC cells after SRC knockdown. Control groups were transfected with non-targeted knockdown shRNA. (D) Immunoblot and grayscale values of SRC protein in EC cells after SRC knockdown. (E) IC50 of doxorubicin in endometrial cancer cells after SRC deletion. LogIC50 of HEC-1A: -6.001, -8.327, -8.574; LogIC50 of Ishikawa: -6.579, -7.916, -7.637. (F) EdU cell proliferation assay and bar graph statistics of endometrial cancer cells treated with DOX after SRC knockdown. (G) Colony formation assay and statistical analysis of doxorubicin-treated endometrial cancer cells with SRC knockdown. (H) Transwell assay to assess changes in invasion ability of doxorubicin-treated EC cells after SRC deletion and bar graph statistics. Y-axis represents the number of cells per FOV. (I) *In vivo* subcutaneous xenograft assay to validate changes in sensitivity of endometrial cancer HEC-1A cells to DOX upon SRC deletion. (J) Statistical analysis of subcutaneous xenograft tumor weight. (K) Statistical analysis of subcutaneous xenograft tumor volume changes. (L) HE and Ki-67 staining of sections from subcutaneous xenograft tumors. The scale bar is 100μm.

To investigate the role of SRC in EC, quantitative real-time polymerase chain reaction was initially employed to validate the mRNA expression levels of SRC within cells (Figure 1A). Western blotting was used to illustrate the SRC protein expression profile (Figure 1B). HEC-1A and Ishikawa cell lines were chosen for subsequent validation studies. The same techniques were used to confirm the mRNA and protein levels of the relevant genes following SRC knockdown in cells (Figure 1C-D). The Cell Counting Kit-8 (CCK-8) assay demonstrated a significant increase in the sensitivity of EC to DOX following SRC knockdown. There was an approximately 1500% increase in killing Ishikawa cells and a 27000% increase in killing HEC-1A cells upon SRC depletion, indicating that SRC loss is a pivotal factor in enhancing the sensitivity of EC cells to DOX (Figure 1E). Subsequent experiments, including 5-ethynyl-2'-deoxyuridine (EdU) assays and colony formation experiments, validated a noticeable reduction in the proliferative capacity of EC cells following DOX treatment after SRC deletion (Figure 1F-G). Wound healing experiments and Transwell assays revealed diminished migration and invasion capabilities of EC cells upon SRC silencing and DOX treatment, suggesting inhibited tumor progression (Figure S1A, Figure 1H). Implantation of prescreened SRC-deficient cells via stable subcutaneous transduction of SRC-deficient lentiviruses in nude mice significantly ameliorated the therapeutic efficacy of DOX (Figure 1I-K). Immunohistochemical staining for Ki-67 demonstrated a reduced tumor proliferation rate following SRC deficiency after DOX treatment (Figure 1L). Therefore, modulating SRC expression is a crucial strategy to enhance the sensitivity of EC cells to DOX. Building on this, we developed an efficient drug delivery system targeting SRC, thereby maximizing its therapeutic potential.

### Constructing the DSNClew Dual-Multivalent Aptamer-Based Targeted Silencing Drug Delivery Platform

Precision medicine is a pivotal strategy for treating numerous diseases, particularly targeted therapies for a variety of cancers. Aptamers effectively recognize multiple proteins on cellular membranes or within the bloodstream, influencing the corresponding biological pathways via interactions with specific receptors. This facilitates precise treatment of diseases. Rolling circle amplification (RCA) is an isothermal enzymatic reaction predicted on a circular template, yielding products with ample binding sites for various chemical entities, which enhances drug loading capacity and programmability^37^. Nucleolin (NCL) is prominently expressed on the cell membrane in many cancers and can selectively bind ligands to modulate cancer progression^38^. Dysregulated nucleolin expression may serve as a prognostic indicator of EC^39^. The transferrin receptor (TFRC, also known as CD71) is upregulated on the surface of various neoplastic cells^40^.

**Figure 2:**
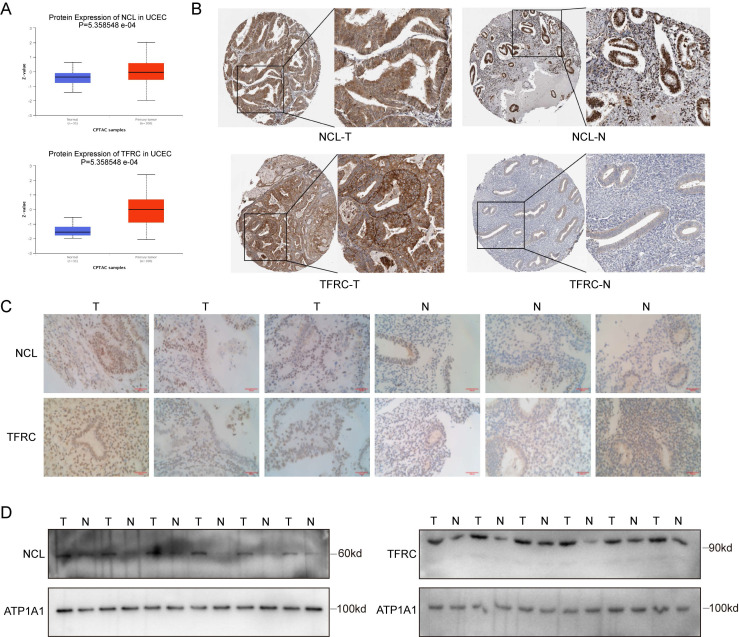
Expression of NCL and TFRC in Endometrial Cancer. (A) Protein expression levels of NCL and TFRC in endometrial cancer (EC) and controls in the CPTAC database. (B) Immunohistochemical staining of NCL and TFRC in endometrial cancer patients and normal endometrial tissues from the HPA database. (C) Immunohistochemical staining of paraffin-embedded sections from clinical endometrial cancer specimens and adjacent non-cancerous specimens. (D) Expression of NCL and TFRC proteins in cell membranes of six pairs of endometrial cancer and adjacent non-cancerous tissue specimens. The scale bar is 100μm.

In this study, we used online repositories (CPTAC) to elucidate the expression patterns of nucleolin and transferrin receptors in EC (Figure 2A). Immunohistochemical staining images of the nucleolin and transferrin receptors obtained from the Human Protein Atlas (HPA) database are presented (Figure 2B). We also conducted immunohistochemical staining of three EC tissues and three normal endometrial tissues, revealing that the tissue epithelium with glandular ducts was extremely positive in tissue specimens from patients with a confirmed diagnosis of EC, whereas in normal endometrial tissues, only some of the staining results in this region were positive. (Figure 2C). Subsequently, epithelial cells were isolated and cultured from six pairs of patients with EC and adjacent tissue epithelial cells. This was followed by membrane protein extraction for western blotting to examine differences in protein levels between the two groups (Figure 2D). Our results indicate elevated nucleolin and transferrin receptor expression on the surface of EC cells. This suggests the potential of nucleolin and transferrin receptors as targets for precise EC cell targeting.

**Figure 3:**
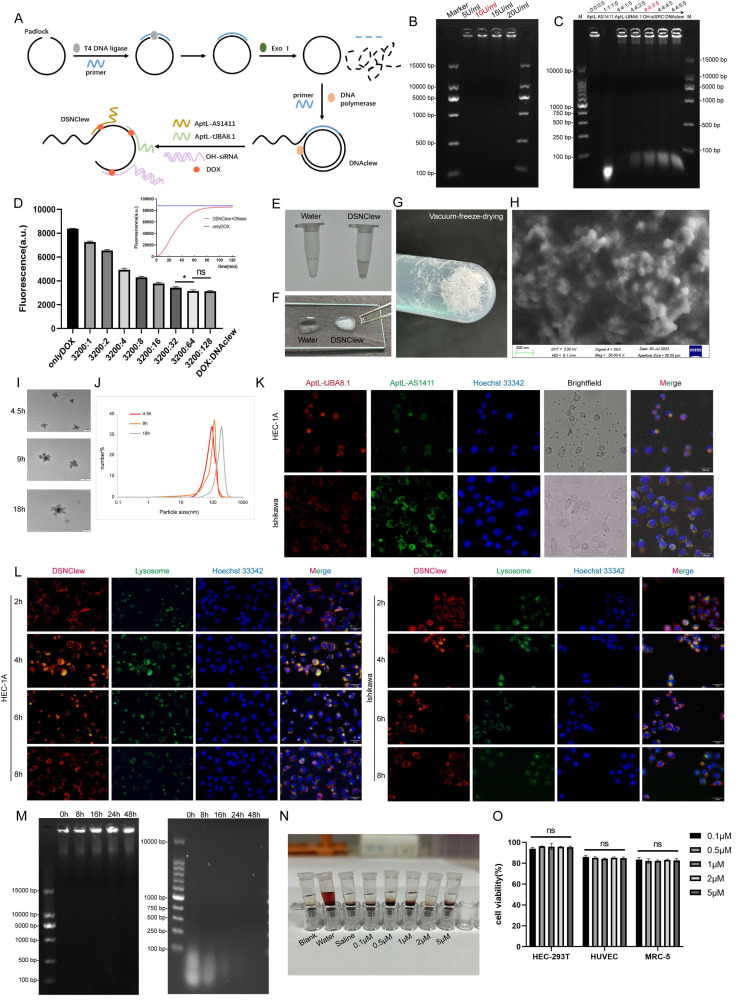
Construction and Characterization of DSNClew. (A) DSNClew synthesis schematic. (B) Exploration of the optimal concentration of Phi29 DNA polymerase. (C) Optimization of the mixing ratio of AptL-AS1411, AptL-tJBA8.1, OH-siSRC, and DNAclew. (D) Adjustment of the drug loading capacity and the drug release efficiency of DSNClew. (E) Characterization of DSNClew in water in test tubes. (F) Comparison of the characteristics of water and DSNClew on glass slides and the extensibility of DSNClew. (G) Morphology of DSNClew after vacuum freeze-drying. (H) Observation of DSNClew morphology using scanning electron microscopy. Electron microscope parameters: EHT=3.00kV, Signal A=SE2, WD=6.1mm, Mag=50.00KX, Aperture Size=30.00μm. (I, J) Transmission electron microscopy observation of DSNClew morphology and particle size distribution produced under different amplification times. The scale bar is 100nm. (K) Localization of DSNClew in endometrial cancer cells using two different aptamers. (L) Observation of DSNClew entry into cells and lysosomal escape phenomenon. (M) Serum stability testing of DSNClew with single-stranded DNA. (N) Hemolysis assay of DSNClew on mouse blood. (O) Toxicity testing of DSNClew at different concentrations in HEC-293T, HUVEC, and MRC-5 cells. The scale bar in K and L are 100μm.

To precisely target EC cells and deliver drugs and gene silencing tools, we leveraged RCA reaction to build a synthesis platform (Figure 3A). We inserted three motifs into the padlock chain to generate a long, single-stranded RCA product with repetitively functional sequences-DNAclew. Serving as a scaffold, DNAclew can bind to four components: tJBA8.1^41^, AS1411^42^, siRNA with an overhang region, and DOX, thus establishing a stable self-assembling multivalent aptamer-modified dual-targeted gene silencing drug delivery platform, DSNClew. tJBA8.1 is a novel high-affinity DNA aptamer with a high overlap at the iron binding site of the transferrin receptor (TFRC) and multiple molecular interactions with the TFRC. tJBA8.1 is a DNA aptamer with a G-quadruplex structure, which selectively binds to tumor cells by targeting nucleolin on the cell surface. Theoretically, upon entry into the bloodstream, DSNClew, guided by aptamers, targets tumor cells overexpressing nucleolin and transferrin receptors on their surfaces, enters cells via endocytosis, and releases siRNA, which binds to the RNA-induced silencing complex in the cytoplasm. This should induce degradation of the corresponding SRC-mRNA. Additionally, it can exert synergistic effects with DOX that enters the cell nucleus, thereby enhancing the sensitivity of EC cells to DOX therapy.

To verify the feasibility of DSNClew, we employed agarose gel electrophoresis to assess the assembled product. The results showed an accumulation of bands at the loading end of the gel, indicating the formation of larger, complex structures, as higher molecular weight assemblies migrate more slowly through the gel matrix. This banding pattern is consistent with the successful synthesis of high-molecular-weight DNAclew complexes, which are retained near the loading site due to their size. This confirms the successful synthesis of DNAclew (Figure 3B). Phi-29 DNA polymerase was used as the DNA polymerase, and T4 DNA ligase served as the ligase at the ends of the padlock chain. We tested four concentrations of Phi-29 DNA polymerase and observed the bands at the up-sampling end of the gel, finding that the brightness of the bands was significantly higher at a concentration of 10 U/ml than that at 5 U/ml (Figure 3B). The brightness of the bands was not significantly enhanced when the concentration was further increased to 15 U/ml and 20 U/ml, and therefore, 10 U/ml was chosen as the optimal concentration for the Phi-29 DNA polymerase to be used. Similarly, we tested the optimal concentration of T4 DNA ligase (Figure S2A). Based on these, we optimized the working time of T4 DNA ligase and Phi-29 DNA polymerase, and the results showed that the brightness of the bands at the up-sampling end reached the plateau value when the working time was set to 3h and 9h, respectively, and the benefit of continuing to increase the working time on the brightness of the bands was not obvious (Figure S2B-C). To achieve DSNClew functionality, DNAclew was assembled with AptL-tJBA8.1, AptL-AS1411, and OH-siSRC, with the assembly proportions adjusted accordingly (Figure 3C, Figure S2D-E). Firstly, the ratio of AptL- AS1411 was gradually increased, and when AptL- AS1411: DNAClew was up-regulated from 0:5 to 4:5, a band appeared at the position of <100bp, which indicated that AptL- AS1411 was excessive, and further increase of the ratio of AptL- AS1411 was not beneficial to improve the function and efficiency of DSNClew. Based on this, similarly, the optimal assembly ratios of AptL-tJBA8.1 and OH-siSRC were continued to be tested. To ensure the highest assembly efficiency for subsequent experiments, the final ratio used was 4:4:3:5. DOX can be inserted into double-stranded 5'-GC-3' or 5'-CG-3' sequences, resulting in fluorescence quenching^43^. To ascertain the drug-loading capacity and release rate of DSNClew, we examined the fluorescence intensity of DOX at different ratios, and found that DOX fluorescence intensity no longer changed when DOX:DNAclew was less than 50:1. Therefore, 50:1 was determined as the maximum drug loading ratio. Subsequently, DOX fluorescence intensity changes were monitored during the reaction of DNase with DSNClew, and the fluorescence intensity plateau value of DSNClew in reaction with DNase was 92% of that in the onlyDOX group, i.e., the release rate of dox was 92%. (Figure 3D). DSNClew formed a viscous, translucent, white liquid with a white mesh-like structure after vacuum freeze-drying (Figure 3E-G). Scanning electron microscopy revealed that DSNClew has a string-beaded structure (Figure 3H). Further observation using transmission electron microscopy revealed a polygonal structure resembling ice crystals with an average diameter of 120 nm. Attempts to extend or shorten the amplification time were made, images of the nanoparticles were captured, and particle size statistics were obtained using transmission electron microscopy, demonstrating that the particle size of DSNClew can be controlled by amplification time (Figure 3I-J).

AptL-tJBA8.1 and AptL-AS1411 can target EC cells; this can help to enhance DSNClew efficacy and reduce adverse reactions. AptL-tJBA8.1 and AptL-AS1411 were labeled with different colored fluorescent tags at their 5' ends, and DNAclew was mixed into EC cells.

Fluorescence microscopy revealed the co-localization of green and red fluorescence-labeled aptamers in EC cells, indicating the ability of AptL-tJBA8.1 and AptL-AS1411 to accurately aim DSNClew at EC cells (Figure 3K). To explore the mechanism of action of DSNClew within cells, we conducted lysosomal escape experiments and found that DSNClew escaped from lysosomes 8 h after administration (Figure 3L). Biological stability is a fundamental requirement for drug carriers. Thus, we tested the stability of DSNClew in the serum compared to that in ssDNA. DSNClew remained stable in the serum for 48 h, whereas ssDNA showed significant degradation after 8 h (Figure 3M). DSNClew was added to fresh mouse blood for hemolysis and compared with positive and negative controls to evaluate its biological toxicity. This confirmed that DSNClew does not significantly promote hemolysis, indicating good blood compatibility (Figure 3N). We also measured DSNClew toxicity at different concentrations in normal cells and found no significant decrease in the viability of HEC-293T, HUVEC, or MRC-5 cells 48 h post-administration (Figure 3O). These experiments demonstrate that DSNClew has good biocompatibility, low toxicity, and potential for systemic applications.

### Validation the Functionality of DSNClew *In Vivo*/*In Vitro*

**Figure 4:**
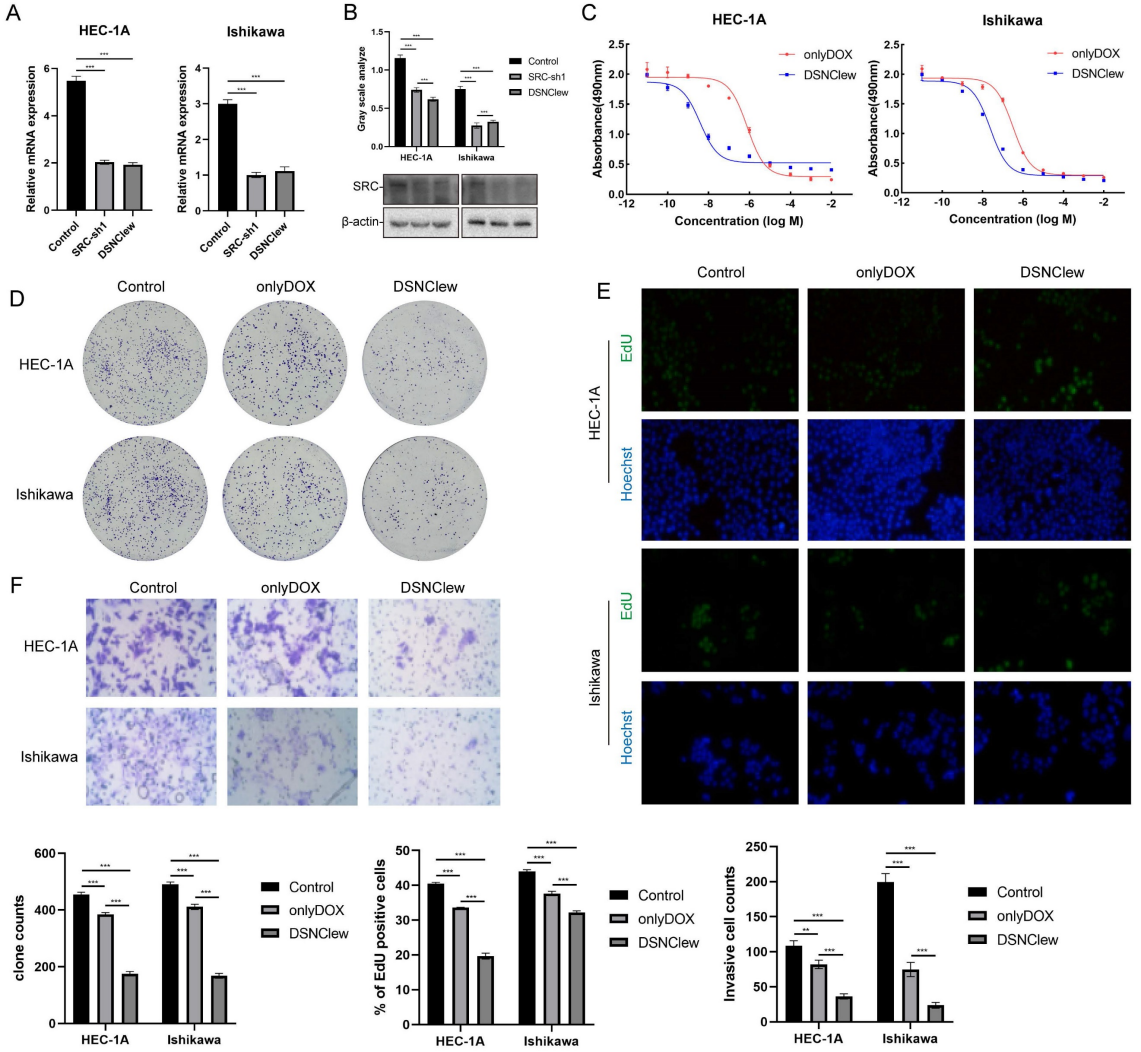
Cellular-Level Validation of DSNClew Function. (A, B) Validation of the silencing component function of DSNClew by qRT-PCR and Western blotting. (C) Changes in IC50 of endometrial cancer cells after DSNClew treatment. LogIC50 of HEC-1A: -6.109, -8.416; LogIC50 of Ishikawa: -6.501, -7.623(D, E) Comparison of colony formation and EdU incorporation assays to assess the proliferative impact of DSNClew treatment versus doxorubicin treatment alone on endometrial cancer cells. (F) Transwell assay to assess the impact of DSNClew treatment on the invasive ability of endometrial cancer cells. Y-axis represents the number of cells per FOV.

**Figure 5:**
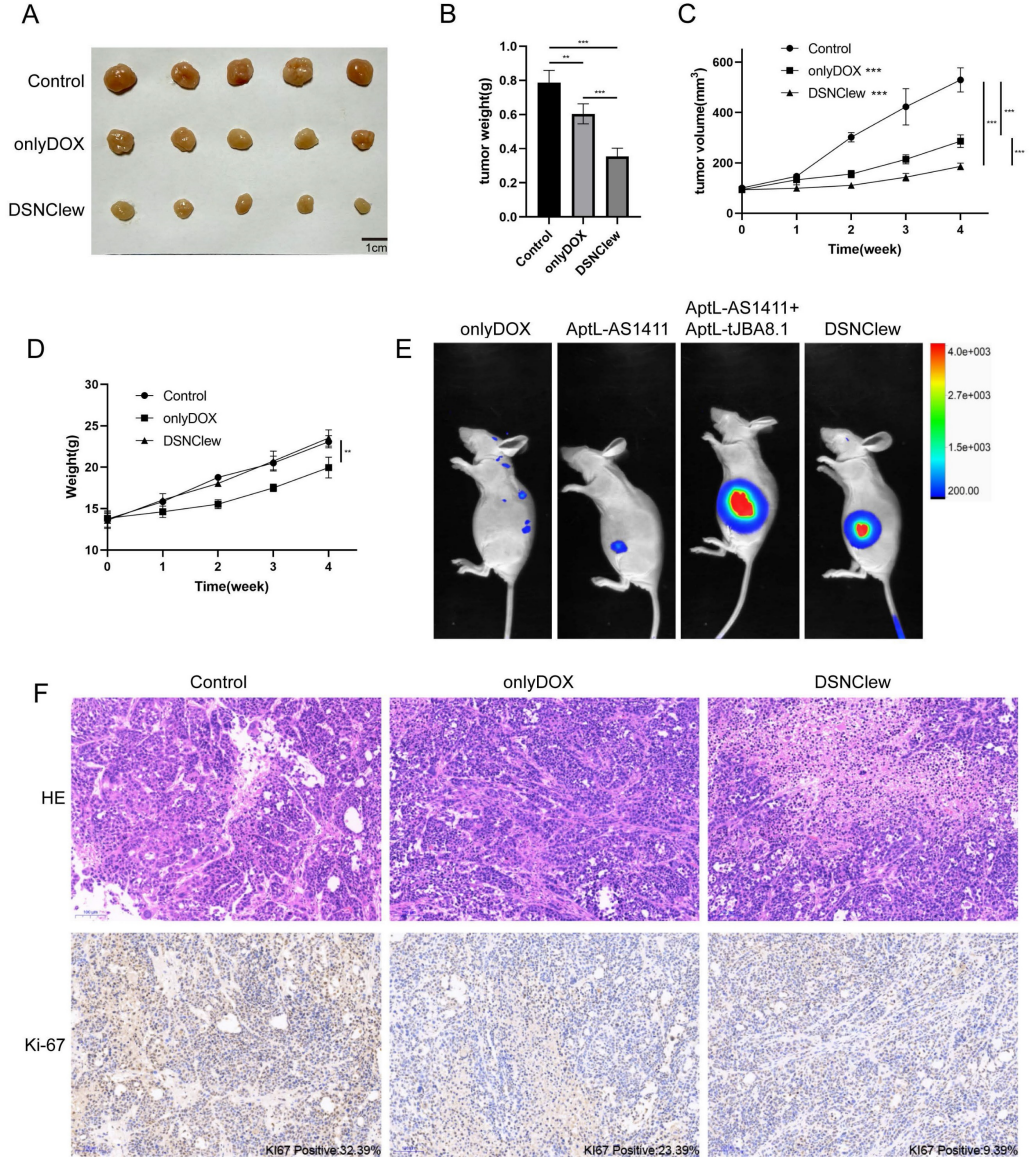
Animal-Level Validation of DSNClew Function. (A) *In vivo* validation of the therapeutic effect of DSNClew on endometrial cancer through subcutaneous xenograft experiments in nude mice. (B) Statistical analysis of subcutaneous xenograft tumor weight. (C) Statistical analysis of subcutaneous xenograft tumor volume changes. (D) Statistics of animal weight changes. (E) Small animal imaging of subcutaneous xenograft tumor models in nude mice to validate the targeting ability of DSNClew. AptL-AS1411 and AptL-tJBA8.1 were labeled with Cy7. The heatmap colors range from blue to red, where blue indicates weaker fluorescence signals, and red indicates stronger fluorescence signals. (F) HE and Ki-67 staining of sections from subcutaneous xenograft tumors. KI67 positivity is marked at the lower right corner of the IHC image. The scale bar is 100μm.

EC cells exhibit poor sensitivity to DOX, as previously confirmed by the absence of SRC significantly enhancing EC cell sensitivity to DOX. We incorporated the OH-siSRC component into DSNClew to silence the SRC gene and validated this at both the RNA and protein levels (Figure 4A-B). We evaluated the sensitivity-regulating function of DSNClew in response to DOX *in vitro*. Co-incubation of DSNClew with cells and testing at different concentrations revealed a significant decrease in IC50 compared to DOX alone, demonstrating that the therapeutic effect of DSNClew on EC cells was significantly higher than that of DOX alone (Figure 4C). Colony formation assays and EdU experiments demonstrated that DSNClew inhibited EC cell proliferation more effectively than DOX alone (Figure 4D-E), whereas wound healing and Transwell assays showed that DSNClew suppressed tumor cell migration and invasion more significantly than DOX alone (Figure S1B, Figure 4F). These results demonstrate the inhibitory effect of DSNClew on EC *in vitro*, surpassing the efficacy of DOX monotherapy. EC cells were subcutaneously implanted in nude mice and DSNClew was administered via tail vein injection. Consequently, DSNClew inhibited subcutaneous tumor growth without affecting animal body weight (Figure 5A-D). Compared with DOX, DSNClew exhibited lower overall toxicity in animals. Imaging of nude mice injected with DSNClew for 4 h revealed the targeting of subcutaneous tumors, with a dual-aptamer signal intensity superior to that of a single aptamer. In contrast, DOX alone did not show significant *in vivo* tumor targeting (Figure 5E). Blood samples collected from the mice for liver and kidney function, markers of myocardial injury, and serum electrolytes testing indicated no significant changes in organ function following DSNClew administration, demonstrating its minimal toxicity* in vivo* (Table 1). We performed HE staining and observation of cardiac tissues and found that mild edema existed in cardiomyocytes of animals using DOX. The nucleus of cardiac muscle fibers in some regions gradually disappeared, the cytoplasmic structure was destroyed, and the transverse stripe became blurred. In contrast, cardiomyocytes using DSNClew did not show such manifestations (Figure S3). Histological examination of fixed and embedded tumor samples, followed by hematoxylin and eosin staining and Ki-67 immunohistochemical staining, confirmed the inhibition of EC proliferation by DSNClew (Figure 5F). Ki-67 mainly used to label cells in the proliferative cycle, the Ki-67 positivity rate of tumor sections in the group using DSNClew showed significantly lower than that of the group using DOX alone and the control group, which indicated inactive cell proliferation, fewer cells in the dividing phase, and less active cell division.

### Underlying Mechanism of the Enhanced EC Cell Sensitivity to DOX Induced by DSNClew

**Figure 6:**
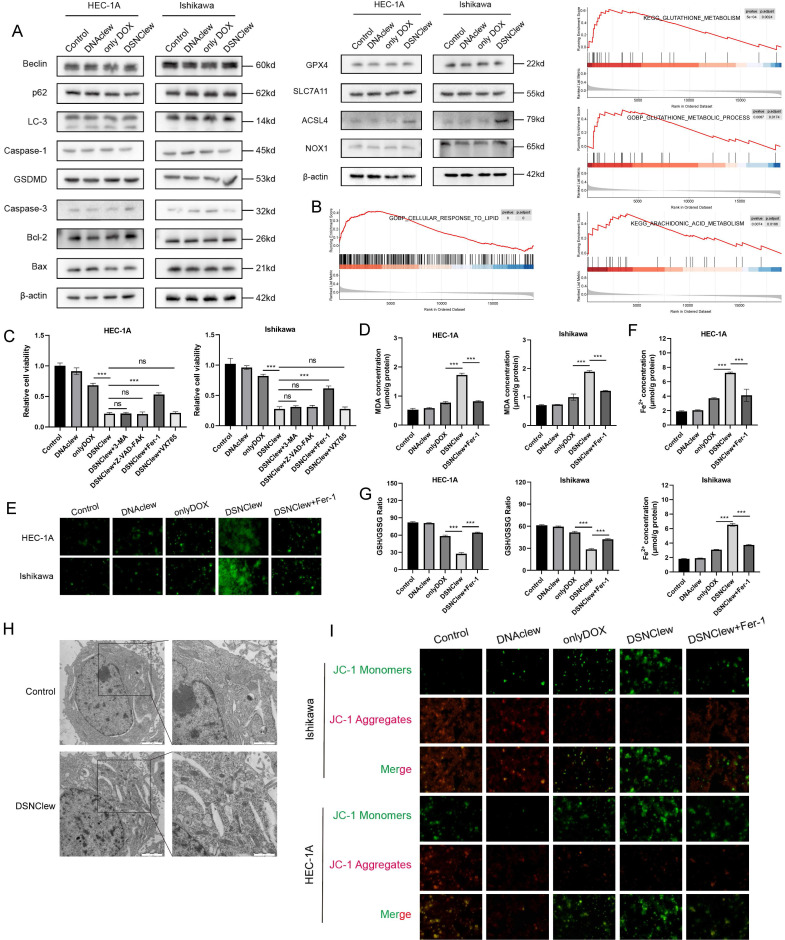
DSNClew Increases Sensitivity of Endometrial Cancer to DOX Through Ferroptosis Pathway. (A) Immunoblotting of proteins related to cellular autophagy, pyroptosis, apoptosis, and ferroptosis. (B) Bioinformatics analysis of SRC-related gene sets using gseaKEGG and gseaGO. (C) Effect of inhibitors of different cell death pathways on cell viability after DSNClew treatment. (D) Measurement of lipid peroxide levels using the MDA assay. (E) Detection of intracellular Fe2+ concentration through iron detection experiments. (F) Reactive oxygen species detection using the fluorescent probe DCFH-DA. (G) Determination of GSH and GSSG levels and calculation of their ratio. (H) Transmission electron microscopy showing mitochondrial morphology. (I) JC-1 assay to detect changes in mitochondrial membrane potential.

DSNClew represents a novel strategy for EC treatment. We investigated the mechanism by which DSNClew influences EC cell sensitivity to DOX. Initially, we delineated various cell death pathways, including autophagy, necroptosis, apoptosis, and ferroptosis, and screened for key molecules (Figure 6A). We observed significant changes in the protein expression of long-chain acyl-CoA synthetase 4 (ACSL4), a hallmark molecule of ferroptosis, after DSNClew administration. Ferroptosis is a regulated form of cell death that depends on iron-mediated oxidative damage, with increased iron accumulation, generation of free radicals, fatty acid availability, and lipid peroxidation, which are crucial inducers^44^. ACSL4, a member of the ACSL family, is key in the biosynthesis and metabolism of fatty acids^45^. It catalyzes arachidonic acid synthesis into arachidonic acid-CoA, which participates in membrane phospholipid synthesis. Ferroptosis involves the formation of peroxides containing polyunsaturated fatty acids (PUFAs) through multistep metabolic reactions, resulting in membrane disruption and cell death^46^. The accumulation of lipid ROS can induce ferroptosis, a process that requires ACSL4 activation of PUFAs^47^. Additionally, GSEA analysis with Kyoto Encyclopedia of Genes and Genomes (KEGG) and Gene Ontology (GO) revealed enriched pathways related to ferroptosis, such as arachidonic acid metabolism, glutathione (GSH) metabolism, and cellular responses to lipid processes (Figure 6B).

To further elucidate the mechanism by which DSNClew enhanced EC cell sensitivity to DOX, we assessed cell viability of different groups using the CCK-8 assay. We observed a significant decrease in cell viability after DSNClew treatment. Upon the addition of apoptosis, autophagy, and necroptosis inhibitors, no notable change in cell viability was observed. Conversely, cell viability was significantly ameliorated when a ferroptosis inhibitor was added. This suggests that the increased sensitivity of tumor cells to DOX following DSNClew treatment is associated with ferroptosis activation (Figure 6C). Furthermore, we evaluated cellular lipid oxidation by measuring malondialdehyde levels. The results reveal elevated lipid oxidation levels after DSNClew treatment, which were reduced upon the addition of a ferroptosis inhibitor (Figure 6D). ROS staining revealed a significant increase in intracellular ROS levels after DSNClew treatment, which decreased upon co-treatment with a ferroptosis inhibitor (Figure 6E). Intracellular iron levels increased after DSNClew treatment but decreased after co-treatment with a ferroptosis inhibitor (Figure 6F). Additionally, we assessed the ratio of GSH to oxidized glutathione (GSSG) and found a decrease in the GSH/GSSG ratio after DSNClew treatment, indicating that DSNClew induced oxidative stress in cells (Figure 6G). Furthermore, scanning electron microscopy revealed morphological changes in cells after DSNClew treatment, including reduced mitochondrial size, increased mitochondrial membrane density, decreased or absent mitochondrial cristae, and fragmented outer mitochondrial membranes; these are characteristics of cells undergoing ferroptosis (Figure 6H). Finally, the JC-1 probe was used to visualize mitochondrial membrane potential. During ferroptosis, oxidative stress decreases the mitochondrial membrane potential, which is detected by the transition of JC-1 fluorescence from red to green (Figure 6I). Our experiments confirmed that DSNClew treatment decreased mitochondrial membrane potential *in vitro*.

**Figure 7:**
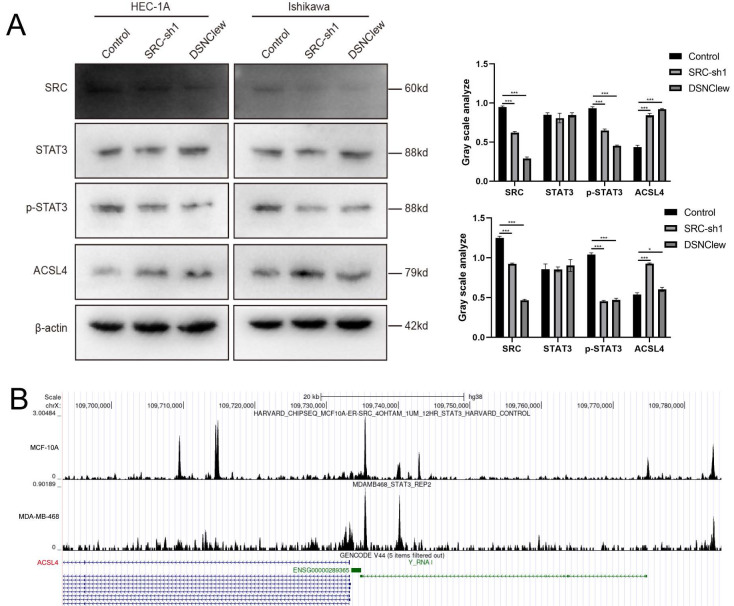
DSNClew Regulates Ferroptosis via the SRC/STAT3/ACSL4 Pathway. (A) Western blotting experiment to verify the effect of SRC knockdown on downstream pathway proteins. (B) CHIP-seq data by using Cistrome DB.

Our experimental findings and previous literature indicate that the loss of SRC is crucial for sensitizing cells to DOX^35^. SRC activity is associated with cellular resistance to ferroptosis, and STAT3 activation depends on SRC kinase activity^48^. Previous studies suggest that STAT3 plays a dual role in transcriptional activation and repression. Therefore, we hypothesized that the increased level of ferroptosis observed after cell treatment with DSNClew is related to ACSL4 regulation by the SRC/STAT3 axis and conducted preliminary verification through protein-level detection experiments^49^ (Figure 7A). In addition, we queried the CHIP-seq data using the Cistrome Data Browser and found the binding site of STAT3 in the promoter region of ACSL4^50^ (Figure 7B). In summary, our results reveal that DSNClew enhances the sensitivity of ECs to ferroptosis through the SRC/STAT3/ACSL4 axis, thereby increasing the responsiveness of EC to DOX.

## Discussion

DNA nanomaterials, including DNA hydrogels and DNA origami, have been extensively explored owing to their excellent biocompatibility, precise molecular programmability, and ease of functionalization. Research on their application in tumor chemotherapy has rapidly advanced. These materials can enhance tumor treatment efficacy through various mechanisms. For example, Wang *et al.* reported DNA nanomaterials that can effectively load chemotherapeutic agents, such as doxorubicin and paclitaxel, and release precisely release them by employing specific depolymerization-triggering mechanisms in response to tumor microenvironment factors such as acidity^51^. This targeted release reduces systemic toxicity and side effects. Moreover, Guo *et al.* developed DNA nanoflowers that can be used in conjunction with immunotherapy and photodynamic therapy, integrating multiple treatment strategies at the tumor site to increase chemotherapy sensitivity through multimodal treatment^52^.

Although DNA nanomaterials have shown significant progress in chemotherapy applications for various solid tumors—such as breast, lung, and liver cancers—research in endometrial cancer is still in the early stages. Current studies mainly focus on basic nanodrug delivery technologies and targeting properties, while the development of DNA nanomaterials specifically targeting endometrial cancer has been relatively limited. This gap has hindered the further application of DNA nanomaterials in precision treatment for endometrial cancer, highlighting the urgent need to develop novel DNA-based nanocarriers to enhance therapeutic efficacy and reduce chemotherapy side effects. In this study, we developed a tool named DSNclew, which utilizes dual aptamer elements to improve the specificity of doxorubicin treatment. Our results demonstrated that DSNclew can accurately target endometrial cancer cells and achieve significant accumulation in animal models. In small animal models, the dual-aptamer strategy exhibited higher signal intensity compared to a single aptamer. Additionally, DSNclew demonstrated low toxicity and reduced adverse effects in organs such as the liver and kidneys, partially addressing the research gap in endometrial cancer and providing a feasible strategy for future research and treatment.

SRC activation is closely related to chemoresistance in various tumors, and its mechanism involves the regulation of cellular signaling pathways. SRC enhances the proliferation and survival of tumor cells by activating pathways such as PI3K/AKT and MAPK, conferring resistance against chemotherapeutic agents^53^.

Additionally, SRC participates in the epithelial-mesenchymal transition (EMT) process, increasing the invasive and migratory capabilities of tumor cells, which is also closely associated with chemoresistance^54^. SRC activation can also upregulate the expression of multidrug resistance genes, promoting the efflux of chemotherapeutic drugs and reducing their accumulation within cells^55^. Although the role of SRC in chemoresistance in tumors such as breast, lung, and colorectal cancers has been extensively studied, its role in endometrial cancer remains relatively unexplored. Current research mainly focuses on the role of SRC in the initiation and progression of endometrial cancer; however, the specific mechanisms of its involvement in chemoresistance have not yet been systematically elucidated^56^-^58^. Addressing this gap will aid in the development of novel targeted therapeutic strategies to improve treatment outcomes in patients with endometrial cancer. DSNclew addresses the issue of low sensitivity to doxorubicin by regulating SRC expression through the incorporation of siRNA, providing a feasible solution at the genetic level. While this approach has been previously applied by Zhang Z *et al.*^59^, the advantage of our study lies in the integration of specificity and therapeutic efficacy, balancing treatment outcomes with reduced adverse effects, while also exploring the mechanisms underlying drug sensitivity regulation.

In addition to its role in chemoresistance, the specific regulatory mechanisms of SRC in ferroptosis in the context of endometrial cancer remain unknown, particularly under conditions of iron homeostasis imbalance in the tumor microenvironment. Furthermore, the specific mechanisms of the involvement of SRC in resistance to chemotherapy and targeted therapy in endometrial cancer, such as EMT and lipid metabolism, remain unclear. Investigating the regulatory potential of SRC in these resistant phenotypes may provide new insights for overcoming resistance. Therefore, combining SRC-targeting strategies with ferroptosis-inducing therapies to enhance treatment sensitivity and reduce resistance risk is an important direction for future research. Our study suggests that the regulatory role of SRC in endometrial cancer may be achieved through the transcriptional repression of ACSL4 by STAT3, which is consistent with the findings of Brown *et al.*^49^. Our experiments further validated this conclusion and clarified that changes in ferroptosis levels resulting from this regulation are critical factors in altering doxorubicin sensitivity in endometrial cancer.

Our study has certain limitations. First, while the screened aptamer targets were not exclusively expressed on the surface of endometrial cancer tumor cells, they were expressed at relatively high levels. This raises a potential off-target risk for DSNclew, although its targeting effect in nude mice was satisfactory; this may have been masked by the shorter drug injection time. Second, we screened DSNClew-induced cell death pathways by detecting signature molecules; however, non-signature molecule-dependent cell death could still occur—such as DNA damage inducing p53-independent apoptosis. This atypical scenario was not considered in our study owing to the substantial workload it would have entailed. Finally, although the duration of our animal experiments was four weeks and the therapeutic effect of DSNClew appeared favorable, the long-term effects and prognosis for the animals remain unknown. Our subsequent studies will aim to address these issues further.

## Conclusions

SRC plays a pivotal role in regulating chemotherapy resistance in various tumors; however, its involvement in EC remains unclear. To address this gap, we successfully engineered a novel drug delivery tool, DSNClew, designed to precisely target EC cells while facilitating gene silencing and drug delivery. Our results confirm that DSNClew significantly enhanced the sensitivity of EC cells to DOX chemotherapy. Further experiments revealed that DSNClew promoted cellular ferroptosis by modulating the SRC-STAT3-ACSL4 axis, thereby augmenting the sensitivity of EC cells to DOX treatment. This discovery lays a crucial theoretical foundation for the development of effective tumor treatment strategies.

## Supplementary Material

The following files are available: Figure S1: Wound healing experiments of HEC-1A and Ishikawa; Figure S2: Optimization of Key Enzyme Parameters for DSNClew Synthesis; Figure S3: HE staining of cardiac tissue, black arrow pointing to edematous tissue; Table S1: Sequences used in this paper.

## Figures and Tables

**Table 1:** Tests of liver and kidney function, serum electrolytes, and cardiac injury markers of animals.

Test		Control	onlyDox	DSNClew
Liver function	ALT (U/L)	30.5±3.4	49.1±2.9	37.3±3.1
	AST (U/L)	89.7±19.4	97.0±22.1	92.9±16.7
	ALP (U/L)	156.1±27.1	197.0±17.2	163.7±22.3
Renal function	BUN (mmol/L)	16.1±2.5	20.1±1.9	17.8±2.2
	CRE (μmol/L)	16.7±2.6	14.7±2.8	15.0±2.4
Cardiac function	CK(U/L)	122.4±20.7	239.7±23.1	169.8±19.4
	LDH(U/L)	223.4±16.9	589.3±31.8	319.7±26.7
Serum electrolytes	K^+^ (mmol/L)	4.2±1.1	4.6±1.4	5.0±0.7
	Na^+^ (mmol/L)	149.6±1.6	155.0±2.3	154.1±2.2
	Cl^-^ (mmol/L)	109.5±1.1	113.2±1.5	111.8±1.9

## Abbreviations

SRC: SRC proto-oncogene
EC: Endometrial cancer
TCGA: The Cancer Genome Atlas
POLE: DNA polymerase epsilon
MSI-H/dMMR: microsatellite instability/high mismatch repair deficiency
CNL: copy-number low
CNH: copy-number high
DOX: Doxorubicin
ROS: reactive oxygen species
ssDNA: single-stranded DNA
DSNClew: Doxorubicin-siSRC-Nanoclew
MDA: Malondialdehyde
CCK-8: Cell Counting Kit-8
EDU: 5-ethynyl-2'-deoxyuridine
RCA: Rolling circle amplification
TFRC: transferrin receptor
NCL: Nucleolin
ACSL4: long-chain acyl-CoA synthetase 4
PUFAs: peroxides containing polyunsaturated fatty acids
KEGG: Kyoto Encyclopedia of Genes and Genomes
GO: Gene Ontology

## References

[1] Siegel RL, Miller KD, Wagle NS, Jemal A (2023). Cancer statistics, 2023. CA Cancer J Clin.

[2] Clarke MA, Devesa SS, Harvey SV, Wentzensen N (2019). Hysterectomy-Corrected Uterine Corpus Cancer Incidence Trends and Differences in Relative Survival Reveal Racial Disparities and Rising Rates of Nonendometrioid Cancers. J Clin Oncol.

[3] Cancer Genome Atlas Research Network, Kandoth C, Schultz N (2013). Integrated genomic characterization of endometrial carcinoma. Nature.

[4] Brooks RA, Fleming GF, Lastra RR (2019). Current recommendations and recent progress in endometrial cancer. CA A Cancer J Clin.

[5] Crosbie EJ, Kitson SJ, McAlpine JN, Mukhopadhyay A, Powell ME, Singh N (2022). Endometrial cancer. The Lancet.

[6] Mutlu L, Harold J, Tymon-Rosario J, Santin AD (2022). Immune checkpoint inhibitors for recurrent endometrial cancer. Expert Rev Anticancer Ther.

[7] Campbell M, Kiss C, Zimmermann M (2023). Childhood Acute Lymphoblastic Leukemia: Results of the Randomized Acute Lymphoblastic Leukemia Intercontinental-Berlin-Frankfurt-Münster 2009 Trial. J Clin Oncol.

[8] Shafei A, El-Bakly W, Sobhy A (2017). A review on the efficacy and toxicity of different doxorubicin nanoparticles for targeted therapy in metastatic breast cancer. Biomed Pharmacother.

[9] Carvalho FS, Burgeiro A, Garcia R, Moreno AJ, Carvalho RA, Oliveira PJ (2014). Doxorubicin-induced cardiotoxicity: from bioenergetic failure and cell death to cardiomyopathy. Med Res Rev.

[10] Finn NA, Findley HW, Kemp ML (2011). A switching mechanism in doxorubicin bioactivation can be exploited to control doxorubicin toxicity. PLoS Comput Biol.

[11] Clementi ME, Giardina B, Di Stasio E, Mordente A, Misiti F (2003). Doxorubicin-derived metabolites induce release of cytochrome C and inhibition of respiration on cardiac isolated mitochondria. Anticancer Res.

[12] Zhang S, Liu X, Bawa-Khalfe T (2012). Identification of the molecular basis of doxorubicin-induced cardiotoxicity. Nat Med.

[13] Järvinen TA, Tanner M, Rantanen V (2000). Amplification and deletion of topoisomerase IIalpha associate with ErbB-2 amplification and affect sensitivity to topoisomerase II inhibitor doxorubicin in breast cancer. Am J Pathol.

[14] van den Heerik ASVM, Horeweg N, de Boer SM, Bosse T, Creutzberg CL (2021). Adjuvant therapy for endometrial cancer in the era of molecular classification: radiotherapy, chemoradiation and novel targets for therapy. Int J Gynecol Cancer.

[15] Mathews C, Lorusso D, Coleman RL, Boklage S, Garside J (2022). An Indirect Comparison of the Efficacy and Safety of Dostarlimab and Doxorubicin for the Treatment of Advanced and Recurrent Endometrial Cancer. Oncologist.

[16] Sajid A, Rahman H, Ambudkar SV (2023). Advances in the structure, mechanism and targeting of chemoresistance-linked ABC transporters. Nat Rev Cancer.

[17] Choi YH, Yu A-M (2014). ABC transporters in multidrug resistance and pharmacokinetics, and strategies for drug development. Curr Pharm Des.

[18] Ma Y, Yang L, Ma J (2017). Rutin attenuates doxorubicin-induced cardiotoxicity via regulating autophagy and apoptosis. Biochim Biophys Acta Mol Basis Dis.

[19] Kciuk M, Gielecińska A, Mujwar S (2023). Doxorubicin-An Agent with Multiple Mechanisms of Anticancer Activity. Cells.

[20] Kong C-Y, Guo Z, Song P (2022). Underlying the Mechanisms of Doxorubicin-Induced Acute Cardiotoxicity: Oxidative Stress and Cell Death. Int J Biol Sci.

[21] Sheibani M, Azizi Y, Shayan M (2022). Doxorubicin-Induced Cardiotoxicity: An Overview on Pre-clinical Therapeutic Approaches. Cardiovasc Toxicol.

[22] Chen T, Ren L, Liu X (2018). DNA Nanotechnology for Cancer Diagnosis and Therapy. Int J Mol Sci.

[23] Wu D, Wang L, Li W, Xu X, Jiang W (2017). DNA nanostructure-based drug delivery nanosystems in cancer therapy. Int J Pharm.

[24] Udomprasert A, Kangsamaksin T (2017). DNA origami applications in cancer therapy. Cancer Sci.

[25] Balakrishnan D, Wilkens GD, Heddle JG (2019). Delivering DNA origami to cells. Nanomedicine (Lond).

[26] Dai L, Liu P, Hu X, Zhao X, Shao G, Tian Y (2021). DNA origami: an outstanding platform for functions in nanophotonics and cancer therapy. Analyst.

[27] Mo F, Jiang K, Zhao D, Wang Y, Song J, Tan W (2021). DNA hydrogel-based gene editing and drug delivery systems. Adv Drug Deliv Rev.

[28] Zhang Q, Lin S, Shi J (2023). Synthesis and Biomedical Applications of DNA Hydrogel. Curr Drug Metab.

[29] Zhu G, Chen X (2018). Aptamer-based targeted therapy. Adv Drug Deliv Rev.

[30] Jabbari A, Sameiyan E, Yaghoobi E (2023). Aptamer-based targeted delivery systems for cancer treatment using DNA origami and DNA nanostructures. Int J Pharm.

[31] Chen C, Zhou S, Cai Y, Tang F (2017). Nucleic acid aptamer application in diagnosis and therapy of colorectal cancer based on cell-SELEX technology. NPJ Precis Oncol.

[32] Peiró G, Ortiz-Martínez F, Gallardo A (2014). Src, a potential target for overcoming trastuzumab resistance in HER2-positive breast carcinoma. Br J Cancer.

[33] van Oosterwijk JG, van Ruler MAJH, Briaire-de Bruijn IH (2013). Src kinases in chondrosarcoma chemoresistance and migration: dasatinib sensitises to doxorubicin in TP53 mutant cells. Br J Cancer.

[34] Lee YS, Choi J-Y, Lee J (2018). TP53-dependence on the effect of doxorubicin and Src inhibitor combination therapy. Tumour Biol.

[35] Indermaur MD, Xiong Y, Kamath SG (2010). Genomic-directed targeted therapy increases endometrial cancer cell sensitivity to doxorubicin. American Journal of Obstetrics and Gynecology.

[36] Zhang L, Rees MC, Bicknell R (1995). The isolation and long-term culture of normal human endometrial epithelium and stroma. Expression of mRNAs for angiogenic polypeptides basally and on oestrogen and progesterone challenges. J Cell Sci.

[37] Mohsen MG, Kool ET (2016). The Discovery of Rolling Circle Amplification and Rolling Circle Transcription. Acc Chem Res.

[38] Chen Z, Xu X (2016). Roles of nucleolin. Saudi Med J.

[39] Lin Q, Ma X, Hu S (2021). Overexpression of Nucleolin is a Potential Prognostic Marker in Endometrial Carcinoma. Cancer Manag Res.

[40] Daniels TR, Delgado T, Rodriguez JA, Helguera G, Penichet ML (2006). The transferrin receptor part I: Biology and targeting with cytotoxic antibodies for the treatment of cancer. Clin Immunol.

[41] Cheng EL, Cardle II, Kacherovsky N (2022). Discovery of a transferrin receptor 1-binding aptamer and its application in cancer cell depletion for adoptive T-cell therapy manufacturing. J Am Chem Soc.

[42] Bates PJ, Reyes-Reyes EM, Malik MT, Murphy EM, O'Toole MG, Trent JO (2017). G-quadruplex oligonucleotide AS1411 as a cancer-targeting agent: Uses and mechanisms. Biochim Biophys Acta Gen Subj.

[43] Lohlamoh W, Soontornworajit B, Rotkrua P (2021). Anti-Proliferative Effect of Doxorubicin-Loaded AS1411 Aptamer on Colorectal Cancer Cell. Asian Pac J Cancer Prev.

[44] Jiang X, Stockwell BR, Conrad M (2021). Ferroptosis: mechanisms, biology and role in disease. Nat Rev Mol Cell Biol.

[45] Doll S, Proneth B, Tyurina YY (2017). ACSL4 dictates ferroptosis sensitivity by shaping cellular lipid composition. Nat Chem Biol.

[46] Chen X, Li J, Kang R, Klionsky DJ, Tang D (2021). Ferroptosis: machinery and regulation. Autophagy.

[47] Stockwell BR, Friedmann Angeli JP, Bayir H (2017). Ferroptosis: A Regulated Cell Death Nexus Linking Metabolism, Redox Biology, and Disease. Cell.

[48] Niu G, Wright KL, Ma Y (2005). Role of Stat3 in regulating p53 expression and function. Mol Cell Biol.

[49] Brown CW, Amante JJ, Goel HL, Mercurio AM (2017). The α6β4 integrin promotes resistance to ferroptosis. J Cell Biol.

[50] Zheng R, Wan C, Mei S (2019). Cistrome Data Browser: expanded datasets and new tools for gene regulatory analysis. Nucleic Acids Res.

[51] Wang J, Zhang T, Li X (2023). DNA Nanobarrel-Based Drug Delivery for Paclitaxel and Doxorubicin. ChemBioChem.

[52] Guo X, Tu P, Wang X (2024). Decomposable Nanoagonists Enable NIR-Elicited cGAS-STING Activation for Tandem-Amplified Photodynamic-Metalloimmunotherapy. Adv Mater.

[53] Kook E, Chun K-S, Kim D-H (2024). Emerging Roles of YES1 in Cancer: The Putative Target in Drug Resistance. Int J Mol Sci.

[54] Sabbah M, Emami S, Redeuilh G (2008). Molecular signature and therapeutic perspective of the epithelial-to-mesenchymal transitions in epithelial cancers. Drug Resist Updat.

[55] Ahn WS, Kim HD, Kim TS, Ahn MJ, Park YJ, Kim J (2023). Phosphorylation of rpS3 by Lyn increases translation of Multi-Drug Resistance (MDR1) gene. BMB Rep.

[56] Marugán C, Sanz-Gómez N, Ortigosa B (2024). TPX2 overexpression promotes sensitivity to dasatinib in breast cancer by activating YAP transcriptional signaling. Mol Oncol.

[57] Diaz-Jimenez A, Ramos M, Helm B (2024). Concurrent inhibition of ALK and SRC kinases disrupts the ALK lung tumor cell proteome. Drug Resist Updat.

[58] Ruiz-Saenz A, Atreya CE, Wang C (2023). A reversible SRC-relayed COX2 inflammatory program drives resistance to BRAF and EGFR inhibition in BRAFV600E colorectal tumors. Nat Cancer.

[59] Zhang Z, Xu D, Wang J (2023). Rolling Circle Amplification-Based DNA Nano-Assembly for Targeted Drug Delivery and Gene Therapy. Biomacromolecules.

