# Supplementary material for: Dual-Multivalent Aptamer-Based Drug Delivery Platform for Targeted SRC Silencing to Enhance Doxorubicin Sensitivity in Endometrial Cancer
Source: Int J Biol Sci. 2024 Oct 28;20(15):5812–30. doi: 10.7150/ijbs.101059 (PMC11628347; doi:10.7150/ijbs.101059)
Supplement: Supplementary file 1 — The following files are available: Figure S1: Wound healing experiments of HEC-1A and Ishikawa; Figure S2: Optimization of Key Enzyme Parameters for DSNClew Synthesis; Figure S3: HE staining of cardiac tissue, black arrow pointing to edematous tissue; Table S1: Sequences used in this paper. [file ijbsv20p5812s1.pdf]

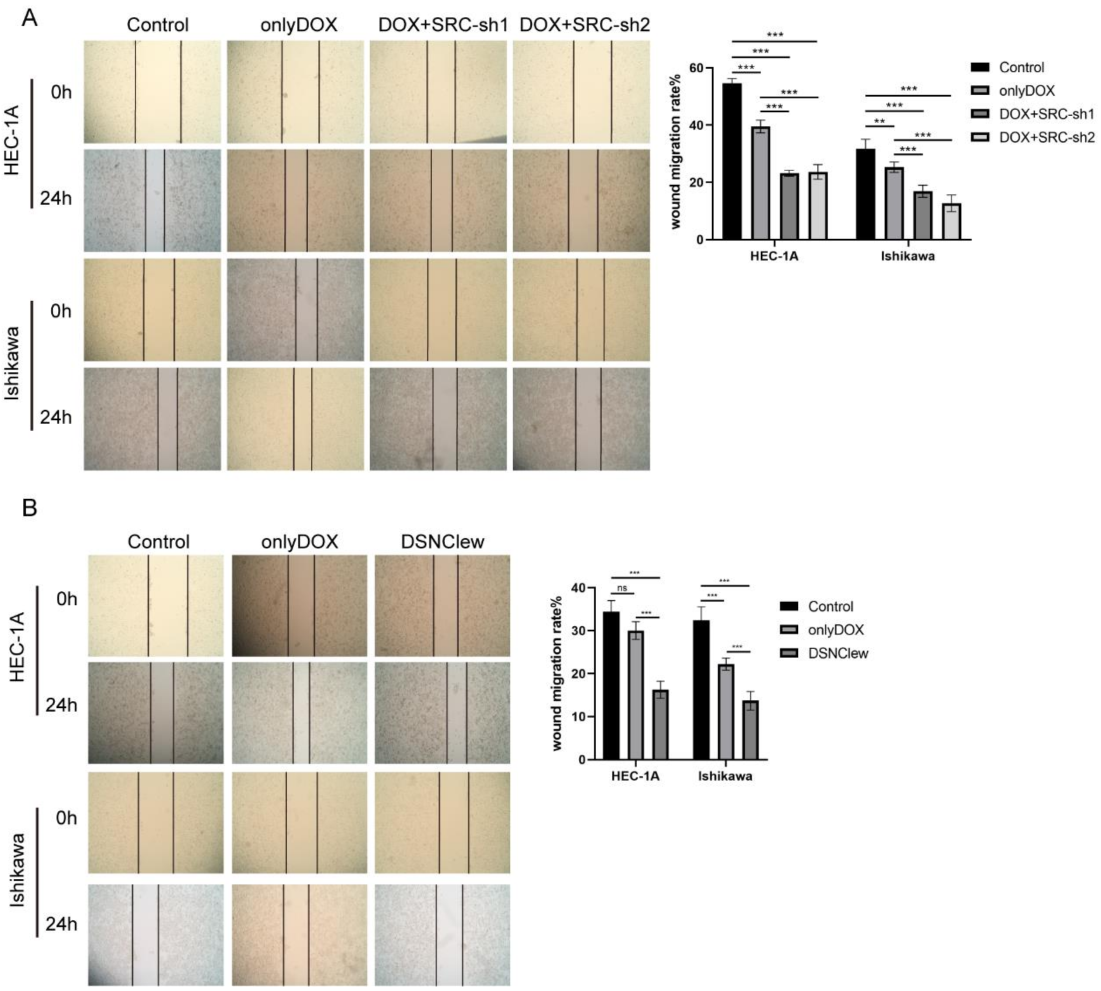

Figure S1. Wound healing experiments of HEC-1A and Ishikawa. (A) Wound healing experiments of knocking down SRC versus DOX alone. (B) Wound healing experiments of DOX alone versus using DSNClew.

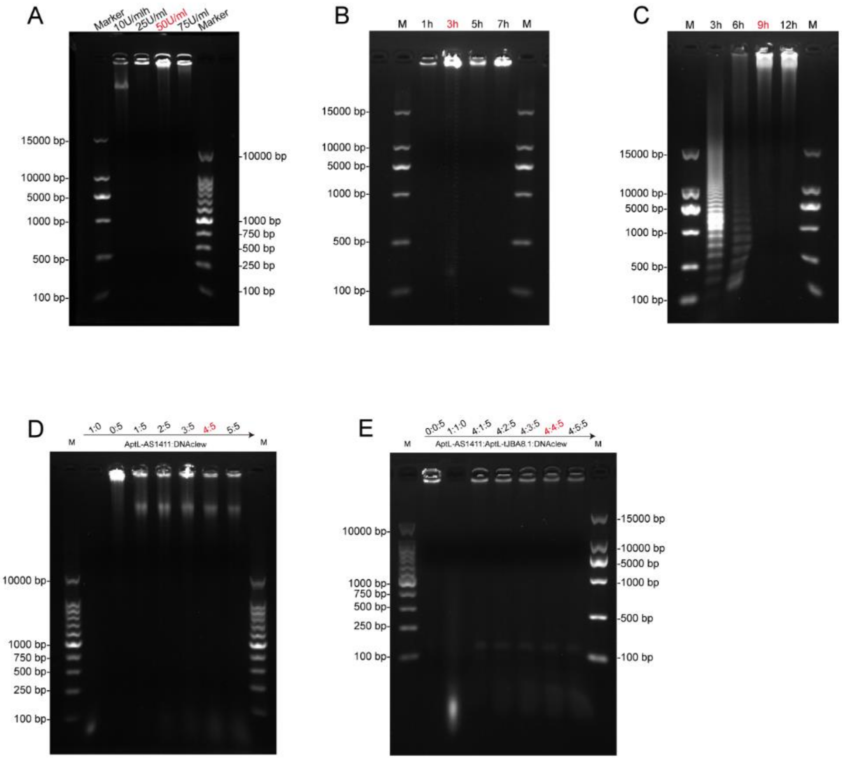

Figure S2. Optimization of Key Enzyme Parameters for DSNClew Synthesis. (A) Different concentrations of T4 DNA ligase. (B) Different incubation times of T4 DNA ligase. (C) Exploration of the optimal incubation time for Phi29 DNA polymerase. (D) Optimization of the mixing ratio of DNAclew and AptL-AS1411. (E) Optimization of the mixing ratios of AptL-AS1411, AptL-tJBA8.1, and DNAclew.

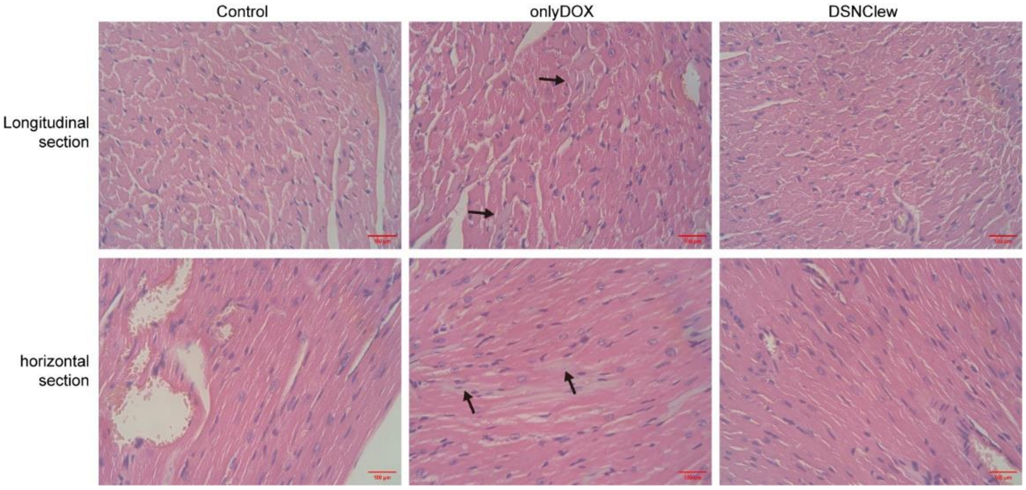

Figure S3. HE staining of cardiac tissue, black arrow pointing to edematous tissue.

Table S1. Sequences used in this paper.

Name	Sequences
Padlock	CTGATAAGCTATCCTAGTCGTAACTTGTAGCATCATTCTCCGAT

	TCCGTTGCCGTGCCTGCAACATCAGT
Primer	TAGCTTATCAGACTGATGTTGC
AptL-AS1411	CATCATTCTCCGATTCCGTTTTTTTGGTGGTGGTGGTTGTGGTGGTGGTGG
AptL-tJBA8.1	GCAGCAGCGTAAAGGGGGTGTTTGTGCGGTGTGGAGTGCGCGTGCTGCTGCTTTTTCTGATAAGCTATCCTAGTCG
SRC-sh1	GAAGAGCAAGCCCAAGGAU
SRC-sh2	GCUUGUGGGUGAUGUUUGA
SRC-sh3	GCUCCAGAUUGUCAACAAC
OH-siSRC-1	AUCCUUGGGCUUGCUCUUCTTGCCGTGCCTGCAACATCAGT
OH-siSRC-2	UCAAACAUCACCCACAAGCTTGCCGTGCCTGCAACATCAGT
OH-siSRC-3	GUUGUUGACAAUCUGGAGCTTGCCGTGCCTGCAACATCAGT
SRC-F	GGCTCCAGATTGTCAACA
SRC-R	GCTTGCGGATCTTGTAGT
β-actin-F	CACCATTGGCAATGAGCGGTTC
β-actin-R	AGGTCTTTGCGGATGTCCACGT
